# A 2D detector array for relative dosimetry and beam steering for FLASH radiotherapy with electrons

**DOI:** 10.1002/mp.17573

**Published:** 2024-12-17

**Authors:** Andreas A. Schönfeld, Jeff Hildreth, Alexandra Bourgouin, Veronika Flatten, Jakub Kozelka, William Simon, Andreas Schüller

**Affiliations:** ^1^ Research and Development Sun Nuclear Corp. Melbourne Florida USA; ^2^ Dosimetry for Radiotherapy Physikalisch‐Technische Bundesanstalt Braunschweig 38116 Germany; ^3^ Present address: Metrology Research Center National Research Council of Canada Ottawa Ontario Canada

**Keywords:** dosimetry, FLASH, quality assurance

## Abstract

**Background:**

FLASH radiotherapy is an emerging treatment modality using ultra‐high dose rate beams. Much effort has been made to develop suitable dosimeters for reference dosimetry, yet the spatial beam characteristics must also be characterized to enable computerized treatment planning, as well as quality control and service of a treatment delivery device. In conventional radiation therapy, this is commonly achieved by beam profile scans in a water phantom using a point detector. In ultra‐high dose rate beams, the delivered dose needed for a set of beam profile scans may exceed the regulatory dose limit specified for a typical treatment room, or degrade components of the scanning system and scanning detector. Point detector scans also cannot quantify the pulse‐to‐pulse stability of a beam profile. Detector arrays can overcome these challenges, but to date, no detector arrays suitable for ultra‐high dose rate beams are commercially available.

**Purpose:**

The study presents the development and characterization of a two‐dimensional detector array for measuring pulse‐resolved spatial fluence distributions in real‐time and temporal structure of intra‐pulse dose rate of ultra‐high pulsed dose rate (UHPDR) electron beams used in FLASH radiotherapy.

**Methods:**

The performance of the SunPoint 1 diode was evaluated by measuring the response of the EDGE Detector in a 20 MeV UHPDR electron beam with a dose per pulse of 0.04 Gy – 6 Gy at a pulse duration of 1 µs or 1.9 µs, and instantaneous dose rates of 0.040 – 3.2 MGy·s^−1^. Based on the findings regarding a suitable signal acquisition technique, a PROFILER 2 detector array made of SunPoint 1 diodes was then modified by minimizing trace resistance, applying a reverse bias, and implementing an RC component to each diode to optimize the transfer of the collected charge during a pulse. The resultant “FLASH Profiler” was then tested in the same UHPDR electron beam.

**Results:**

The FLASH Profiler exhibited a linear response within ± 3% deviation over the investigated dose per pulse range. The FLASH Profiler array showed good agreement with the absolute dose measured using a flashDiamond point detector and an integrating current transformer for dose‐per‐pulse values of up to 6 Gy. The FLASH Profiler was able to measure lateral beam profiles in real‐time and on a single‐pulse basis. The ability to capture and display the profiles during steering of UHPDR beams was demonstrated. The SunPoint 1 diode was able to measure the pulse duration and the intra‐pulse dose rate with a time resolution of 4 ns.

**Conclusion:**

The FLASH Profiler could be used for characterizing UHPDR electron beams and facilitating quality control and beam steering service of electron FLASH irradiators.

## 1 INTRODUCTION

FLASH radiation therapy is an emerging treatment modality using ultra‐high dose rate beams which have been reported to enhance healthy tissue sparing.^1^, ^2^, ^3^, ^4^, ^5^, ^6^, ^7^, ^8^, ^9^, ^10^ Research in this field is often conducted on clinical linear accelerators adopted for ultra‐high pulsed dose rates^11^, ^12^, ^13^, ^14^ (UHPDR) and the first UHPDR treatment delivery devices (TDD) using electron beams are being adopted clinically.^15^, ^16^, ^17^ Due to the dose‐per‐pulse exceeding those of conventional external beam radiation therapy by orders of magnitude, common ionization chambers suffer from significantly reduced charge collection efficiency.^18^, ^19^, ^20^, ^21^, ^22^, ^23^, ^24^, ^25^ In the scope of the EURAMET project UHDpulse,^26^ much effort has been made to develop suitable point dosimeters for reference dosimetry in UHPDR beams^27^, ^28^ and first successes have been reported with semiconductors,^29^, ^30^, ^31^, ^32^, ^33^, ^34^, ^35^ scintillators^36^, ^37^ and calorimeters.^38^, ^39^, ^40^, ^41^

While point detectors enable output calibration of a UHPDR TDD, the spatial characteristics of the emitted beam must be characterized to enable computerized treatment planning, as well as quality control and service of the TDD. The spatial fluence distribution of a conventional radiation therapy beam is commonly characterized by scanning lateral beam profiles with a point detector in a water phantom as described in well‐established dosimetry protocols.^42^, ^43^, ^44^, ^45^, ^46^ In UHPDR beams, the delivered dose for a set of beam profile scans may exceed the regulatory dose limit specified for a typical treatment room, or degrade components of the scanning system and scanning detector.^47^

Conventional beam profile scans are performed under the assumption that the beam shape is constant throughout the duration of the scan and that the typical ramp‐up behavior of a well‐adjusted TDD has an insignificant impact on a typical beam delivery. Since UHPDR beams deliver high doses during the first few pulses of a beam and the total delivery time is short, the temporal behavior of a beam delivery is important to understand. Point detector scans cannot quantify the pulse‐to‐pulse stability of a beam profile.

While detector arrays have addressed these challenges in conventional radiation therapy since the introduction of the MapCHECK diode array,^48^ no detector arrays have been available for UHPDR beams. This study characterizes SunPoint 1 silicon diodes built in the EDGE Detector (Sun Nuclear Corp., Melbourne, FL, USA) in UHPDR electron beams. The findings regarding a suitable signal acquisition technique served in the modification of a PROFILER 2 detector array (also Sun Nuclear Corp.), which utilizes the same diode type. The detector array resulting from this modification hereon called FLASH Profiler is capable of real‐time, pulse‐by‐pulse beam profile measurements of UHPDR electron beams.

## 2 MATERIAL AND METHODS

### 2.1 FLASH irradiation facility

The measured data was collected at the Metrological Linear Accelerator Facility of the German National Metrology Institute Physikalisch‐Technische Bundesanstalt (PTB) in Braunschweig.^49^, ^50^ The pulse repetition frequency of the 20 MeV beam was set to $f_{p}=$ 5 Hz, the pulse length $t_{p}$ = 1 µs or 1.9 µs, and the pulse charge was varied from 30 to 235 nC by changing the width of a slit diaphragm (2 – 10 mm) at the beginning of the beamline. 75 pulses were delivered per beam setting.

A Bergoz integrating current transformer (ICT; in‐flange version, turns ratio 50:1) located in the beamline provided a signal proportional to the beam output.^49^ Thus, the ICT signal could be calibrated in terms of absorbed dose to water D at a reference point in a phantom. The calibration of the ICT signal was performed using a flashDiamond (fD) detector^28^, ^30^, ^31^ (SN 7610 by PTW Freiburg, Germany), which was previously calibrated with PTB's alanine dosimetry system. The fD detector was positioned in a 30 × 30 × 30 cm^3^ water phantom on the horizontal beam axis at the maximum of the percentage depth dose curve (water equivalent reference depth $d_{\mathrm{ref}}=$ 30.2 mm). The mean ionization charge collected per pulse was converted to the corresponding dose per pulse, $D_{p}$, by applying the predetermined calibration factor of 0.2498 nC·Gy^−1^.

The water phantom was set up at a distance of 90 cm from the beam exit window. The fD was used with a Keithley 616 electrometer operated in a current mode in conjunction with a 33 nF capacitor to reduce the voltage on the signal cable sufficiently to ensure that the diode in the fD detector remains nonconductive. Further details on the fD are described in previous publications.^30^, ^31^, ^33^, ^50^

By optionally positioning three different aluminum scattering discs of 1 mm, 2 mm, or 6 mm thickness in addition to the 100 µm copper exit window of the beamline, and by the variation of the charge per pulse, $D_{p}$ could be varied from 0.04 – 6 Gy at $d_{\mathrm{ref}}$ on the beam axis. For each setup with respect to the choice of scattering disc and pulse duration, the beam pulse charge was varied and recorded by means of the ICT. $D_{p}$ at $d_{\mathrm{ref}}$ was simultaneously measured by means of the calibrated fD. A calibration function was then fitted to the data sets, which allows to convert the measured ICT signal to $D_{p}$ at $d_{\mathrm{ref}}$. This calibration procedure has been successfully applied in numerous studies on detector response in UHPDR electron beams.^19^, ^20^, ^21^, ^24^, ^30^, ^34^, ^39^ A more detailed description of the beam monitor calibration can be found in Kranzer et al.^19^, ^30^ The only difference in this study is the use of the fD as a transfer reference instead of direct ICT calibration with alanine. The agreement with the results of a direct alanine calibration is within ± 0.5% over the entire DPP range (see Figure 4 in Subiel et al.^28^).

Since the ICT records the pulse charge of each individual pulse, using this calibration function with the measured pulse charge permits the analysis and correction of any pulse‐to‐pulse output variation during measurements.

Due to the roughly rectangular time structure of the pulse, the corresponding instantaneous dose rates of 0.040 – 3.2 MGy·s^−1^ can be approximated as average intra‐pulse dose rates using the pulse lengths as measured with an oscilloscope connected to the ICT.

### 2.2 EDGE detector

**FIGURE 1:**
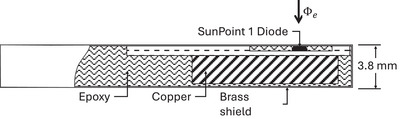
Schematic cross‐section of the EDGE Detector (Sun Nuclear Corp.) assembly including a SunPoint 1 silicon diode. The entrance window is at the top of the diode. $\Phi_{e}$ indicates the incident electron fluence. Measures are not to scale.

The EDGE Detector is designed for water phantom scanning of small to medium photon beam field sizes in conventional external beam radiation therapy. Its suitability for UHPDR electron beam measurements has recently been demonstrated.^29^, ^35^ The EDGE Detector contains a single SunPoint 1 silicon diode with an active area of 0.8 × 0.8 mm^2^. A simplified cross‐sectional drawing is shown in Figure 1.

The SunPoint 1 diode's construction allows its installation in detector arrays, which has been demonstrated in widely used devices such as the MapCHECK 1 and 2, ArcCHECK, Profiler 1 and 2, TomoDose and SRS Profiler (all Sun Nuclear Corp.). This study uses the EDGE Detector to modify electrical components connected to the SunPoint 1 diode, characterize the impact on the diode's performance in UHPDR electron beams, and transfer these changes to a modified Profiler 2 diode array.

The sensitive volume of an EDGE Detector was positioned at $d_{\mathrm{ref}}$ and its response was characterized by varying values of $D_{p}$. To evaluate the response dependence on the instantaneous dose rate, two different pulse durations of $t_{p}=$ 1 µs and $t_{p}=$ 1.9 µs were used while the pulse magnitude was varied. The detector was connected to a Keithley 616 electrometer operated in current mode.

**FIGURE 2:**
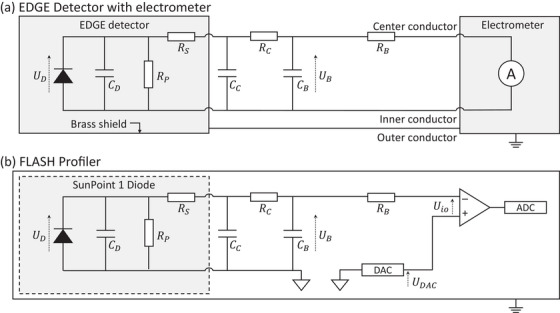
(a) The circuit diagram of an EDGE Detector connected to a current meter with a triaxial cable. The cable capacitance $C_{C}$ and the cable resistance $R_{C}$ were modified by using different cable lengths. An RC “buffer” component was added with varying values of $R_{B}$ and $C_{B}$. The parallel resistance $R_{P}$ of the EDGE detector representing recombination processes is of the order of $8\cdot 10^{8}\Omega$ and can be neglected for the purpose of this study. The EDGE Detector's capacitance $C_{D}$ is on the order of 40 pF. An analogue representation presentation has been published for diamond detectors.^30^ (b) The circuit diagram of a SunPoint 1 diode, i.e. the same diode type as used in the EDGE Detector, as used in the FLASH Profiler. The modifications with respect to a Profiler 2 Detector array include the addition of a DAC (digital‐to‐analog‐converter) to apply a reverse bias voltage to the diode by adding $U_{\mathrm{DAC}}=\;0.6$ V to the input offset voltage $U_{\mathrm{io}}$, as well as of an RC component with $C_{B}=$ 330 nF and $R_{B}=$ 330 Ω. ADC, analog‐to‐digital‐converter.

A real EDGE Detector's circuit is represented in Figure 2a. The EDGE Detector part consists of an ideal diode, a substitute diode capacitance $C_{D}$, a substitute parallel resistance $R_{P}$, and a substitute series resistance $R_{S}$ model. The charge transfer from the EDGE detector to the electrometer was slowed using an RC component (“buffer”) $R_{B}$ and $C_{B}$. The temporary storage of charge on $C_{B}$ and the resistor $R_{B}$ serve to reduce the required power output from the electrometer's operational amplifier, while $R_{B}$ also prevents oscillation between the electrometer's operational amplifier and the capacitor $C_{B}$. Two cable configurations were used where $R_{C}$ was either 2 Ω or 25 Ω and $C_{B}$ was varied from 33 to 2120 nF. The resistance value of $R_{S}+R_{C}$, as well as the capacitance $C_{B}+C_{C}$, determine the voltage $U_{D}$ on the diode when a certain amount of charge is generated during a dose pulse. Once $U_{D}$ exceeds the diode's forward junction voltage, the diode becomes conductive and its response is nonlinear since a portion of the collected charge is lost. The effect can be restricted by applying a reverse bias voltage.^51^ During the EDGE Detector study, no reverse bias was applied.

### 2.3 EDGE detector — Time structure of the pulse response

Using the setup described above, the voltage increase on the capacitor $C_{B}$ was measured with an oscilloscope during a pulse with a temporal resolution of 4 ns. The measurement was repeated with different capacitor sizes $C_{B}$ ranging from 33 to 2120 nF, where the cable's resistance was $R_{C}=$ 1.3 Ω and the cable's capacitance $C_{C}$ was 3.5 nF.

In a further setup, the EDGE Detector and the ICT were connected to an oscilloscope with 4 ns temporal resolution to simultaneously measure the beam current and ionization current from the EDGE detector via the voltage drop over a measuring resistor of $R_{M}=$ 16.7 Ω. This increased the series resistance to $R_{S}+R_{M}$ ≈ 19 Ω. The measured currents were converted to dose rate using the respective response characteristics determined in the previous section.

### 2.4 EDGE detector — Current‐voltage characteristic

The current‐voltage characteristic of the SunPoint 1 Diode was measured with a universal 16‐bit data acquisition board that generates a voltage ramp and derives the diode current from the voltage drop across a shunt resistor.

### 2.5 FLASH profiler

The Profiler 2 detector array (Sun Nuclear Corp.) consists of 139 SunPoint 1 diodes placed in two intersecting lines to simultaneously measure lateral beam profiles in *x*‐ and *y*‐directions. The center‐to‐center spacing of the diodes is 4 mm. The Profiler 2 is controlled with the legacy Profiler Software version 3.0.1. capable of capturing, processing, and displaying beam measurements with a sampling frequency of 10 Hz.

While Profiler 2 is no longer available through commercial channels, some units were modified to reduce $R_{C}$ to less than 1 Ω, where the actual value depends on the length of the electrical traces. A reverse bias voltage of $U_{DAC}+U_{io}=$ 0.6 V and an RC component with $C_{B}=\;330$ nF and $R_{B}=\;330\;\Omega$ were implemented. A circuit diagram including the modifications is represented in Figure 2b. Since the resolution of the AD converter is effectively limited to 14.7 bit, the RC component was optimized for the FLASH Profiler to measure in the range of 1 – ultra‐high pulsed dose rate 8 Gy per pulse, resulting in a reduced bit resolution and greater apparent noise for dose per pulse values of less than 1 Gy.

In the setup, the electronics of the array were protected with additional shielding material, since the electron beam is not collimated. A Wide Field Array Calibration^52^ was performed with the 6 mm aluminum scatter disc in place which provides a flat beam profile. The same series of beams used for the EDGE Detector characterization was delivered to the FLASH Profiler with its plane of measurement placed at a water‐equivalent depth of 33 mm. Lateral beam profiles were measured with the fD point‐by‐point at the same water equivalent depth as reference.

A beam steering process of a UHPDR beam was simulated by altering the current of a steering magnet near the beam exit window during a simultaneous FLASH Profiler measurement.

## 3 RESULTS

**FIGURE 3:**
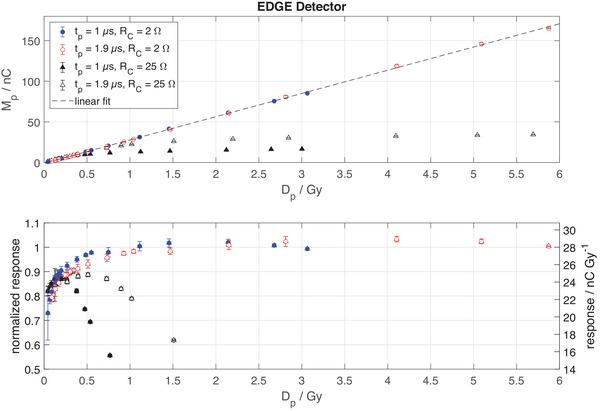
The top panel shows the measured charge per pulse $M_{p}$ of the EDGE Detector in ultra‐high pulsed dose rate electron beams as a function of the dose per pulse $D_{p}$. Two different pulse lengths $t_{p}$ were delivered (solid and open symbols) and two different cable resistances $R_{C}$ were realized (circles and triangles) with a capacitor $C_{B}=$ 2120 nF. The bottom panel shows the same data normalized to the response at 1 Gy per pulse, that is 28 nC/Gy, where the left *y*‐axis shows a relative scale and the right *y*‐axis is an absolute scale of the response. Error bars indicate the variance of measurement points and may be hidden by symbols.

Using a cable with a cable resistance $R_{C}$ = 2 Ω and an added capacitance $C_{B}=$ 2120 nF, the response of the EDGE Detector is linear within ± 3% for UHPDR electron beams with $1\;\mathrm{Gy}\;<\;D_{p}\;<\;6\;\mathrm{Gy}$ (Figure 3). The response is nonlinear with a deviation from linearity of up to 20% for $D_{p}\;<$ 1 Gy.

An increased cable resistance $R_{C}$ = 25 Ω causes the detector response to drop sharply at a dose per pulse $D_{p}$ exceeding about 0.4 Gy for a pulse length $t_{p}=$ 1.9 µs and at $D_{p}$ exceeding about 0.2 Gy for $t_{p}=$ 1 µs. In both cases the instantaneous dose rate $\frac{\delta D_{p}}{\delta t_{p}}$ is about $4\cdot 10^{5}$ Gy·s^−1^ and the resulting voltage on the diode $U_{D,p}=\left(R_{S}+R_{C}\right)\cdot I_{p}\;\approx \;0.35\;\pm \;0.1$ V, where $I_{p}$ is the ionization current during the pulse. This method only allows a rough estimation of $U_{D,p}$, due to uncertainties associated with the measurement of $R_{S}+R_{C}$ and their effect on the diode response (bottom panel of Figure 3) necessary to convert the measured instantaneous dose rate to ionization current.

**FIGURE 4:**
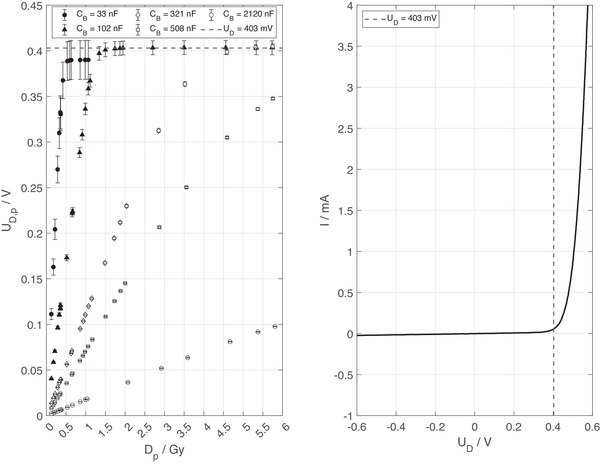
Left: The voltage $U_{D,p}$ on the EDGE Detector diode during a delivered pulse as a function of the dose per pulse $D_{p}$, where $U_{D,p}$ is set by the capacitor size $C_{C}+C_{B}$ for a given dose per pulse. Error bars indicate the variance of measurement points and may be hidden by symbols. Right: The current‐voltage characteristic of the SunPoint 1 diode. The dashed line corresponds to a voltage of $U_{D}=$ 0.4 V in both panels.

**FIGURE 5:**
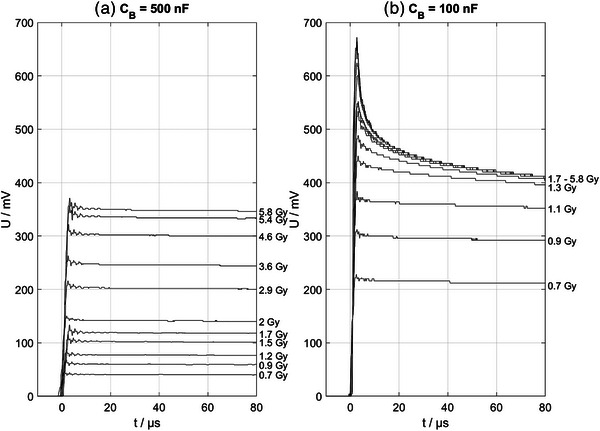
Voltage on the capacitor $C_{B}$ as function of time after pulse start measured with an oscilloscope for varying doses per pulse for $C_{B}=500$ nF in panel (a) and $C_{B}=100$ nF in panel (b). The smaller capacitor in panel (b) increased the voltage $U_{D}$ on the diode proportionally, causing fast discharge through the diode when $U_{D}$ exceeds 0.4 V where diode is conducting. The dose per pulse values were derived from the ICT measurements. Only a selection of annotations was added to panel (b), due to stack‐up. The dose per pulse values, however, correspond to those labeled in panel (a).

Therefore, the observed diode voltage dependence of the response was further investigated by varying the capacitance $C_{B}$, while using the short cable with a cable resistance $R_{C}=\;2\;\Omega$, which affects the voltage on the diode with $U_{D,p}=\frac{M_{p}}{C_{B}+C_{C}}$ for a given charge $M_{p}$ collected during a pulse. As illustrated in the left panel of Figure 4, the voltage $U_{D,p}$ at the diode never exceeds about 0.4 V, which corresponds to the forward junction voltage of the SunPoint 1 Diode (right panel of Figure 4). The underlying effect was demonstrated by measuring the voltage $U_{B}$ on the capacitor $C_{B}$, and thus the voltage on the diode $U_{D}$, as a function of time for different dose‐per‐pulse values (Figure 5). The charge‐up of the capacitor by the diode's ionization current during the 1.9 µs pulse is visualized. Since the electrometer needs several milliseconds to discharge the capacitor, the voltage on the capacitor does not change significantly during the displayed first 80 µs after the pulse. A larger capacitance of $C_{B}=$ 500 nF results in proportionally lower voltages on the diode for a given charge induced by a pulse. While the voltage on the larger capacitor ($C_{B}=$ 500 nF) does not exceed about 0.4 V for the tested range of $D_{p}$ (left panel of Figure 5), the same measurements with a smaller capacitor ($C_{B}=$ 100 nF) reveal a fast discharge through the diode for all pulses causing a voltage exceeding about 0.4 V (right panel of Figure 5). The discharge through the diode stops when the voltage reaches about 0.4 V and the remaining charge is collected by the electrometer, which also explains the plateau of the response values seen for the higher cable resistance in the top panel of Figure 3.

**FIGURE 6:**
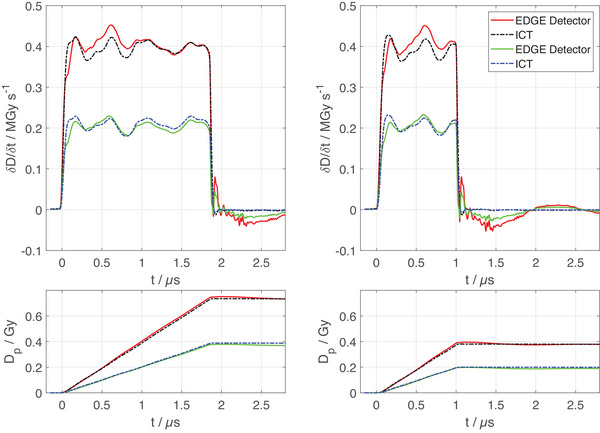
Top panels: The intra‐pulse dose rate measured with an EDGE Detector for nominal values of $t_{p}$ = 1.9 µs (left) and $t_{p}$ = 1 µs (right) in comparison with the measurement using the ICT. The corresponding bottom panels show the accumulated dose measured over the duration of the pulse. The measurements were taken with $R_{S}+R_{M}\;\approx$ 19 Ω. Both devices measured pulse lengths of 1.85 µs (left) and 0.97 µs (right).

The intra‐pulse dose rate as measured with the EDGE Detector at $R_{S}+R_{M}$ ≈ 19 Ω and the ICT is shown in Figure 6 for $D_{p}$ values of 0.20, 0.39, and 0.73 Gy. The EDGE Detector signal matches that of the ICT. Both devices measured the pulse lengths at 1.85 µs and 0.97 µs. In this measurement setup, larger doses per pulse would lead to the previously observed nonlinearity of response (Figure 3), due to the necessary measuring resistor $R_{M}$.

**FIGURE 7:**
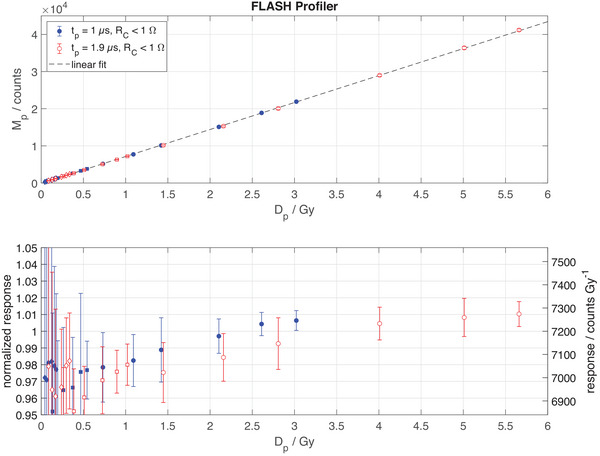
The top panel shows the measured signal per pulse $M_{p}$ of the central diode of the FLASH Profiler in ultra‐high pulsed dose rate electron beams as a function of the dose per pulse $D_{p}$. Two different pulse lengths $t_{p}$ were delivered (solid and open symbols). The bottom panel shows the same data normalized to the response at 1 Gy per pulse, that is 7200 counts/Gy, where the left *y*‐axis shows a relative scale and the right *y*‐axis is an absolute scale of the response. Error bars indicate the variance of measurement points and may be hidden by symbols. The increased noise at $D_{p}<1$ Gy is due to the dynamic range of the AD converter being optimized for $D_{p}>1$ Gy.

The nonlinearity of response observed with the EDGE Detector setup for $D_{p}\;<1$ Gy in Figure 3 was reduced to < 3 % in case of the FLASH Profiler (Figure 7). For $D_{p}\;>\;1$ Gy, the response is linear within ± 2%. No dependence of the response on the pulse length at equal instantaneous dose rate is observed.

**FIGURE 8:**
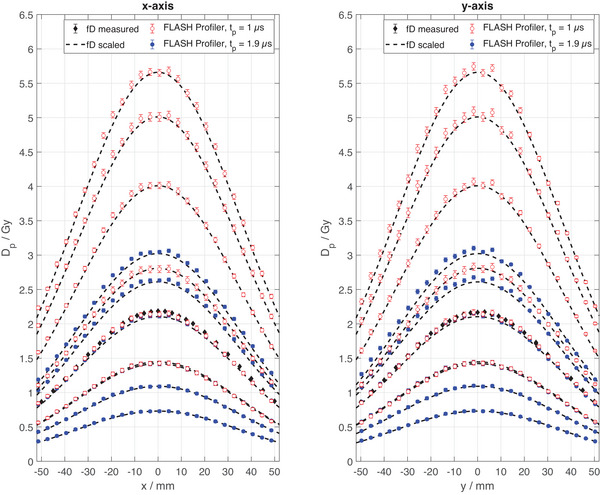
Lateral beam profiles measured with the FLASH Profiler in comparison to stepwise lateral beam profile scans measured with the flashDiamond. Red open symbols and blue solid symbols represent pulse lengths of $t_{p}$ = 1.9 µs and $t_{p}$ = 1 µs. Dashed lines represent scaled fD profile scans based on the $D_{p}$ measured by the calibrated ICT.

Lateral beam profiles were measured with the FLASH Profiler in a setup with no aluminum scatter disc for $D_{p}$ up to 5.5 Gy and compared to lateral beam profile scans measured stepwise with the fD (Figure 8). Since scattering at the beam exit window is, in good approximation, independent from charge per beam pulse, the full‐width‐half‐maximum (FWHM) of the radiation field is nearly constant for a given setup.^50^ Due to the significant time and dose which are necessary for stepwise beam profile scans with the fD, the profiles were measured only for $D_{p}=$ 2.1 Gy and scaled to other values of $D_{p}$ based on the ICT signal recorded simultaneously with the FLASH Profiler. The profiles measured with the FLASH Profiler and the fD agree within measurement uncertainty for both pulse lengths.

**FIGURE 9:**
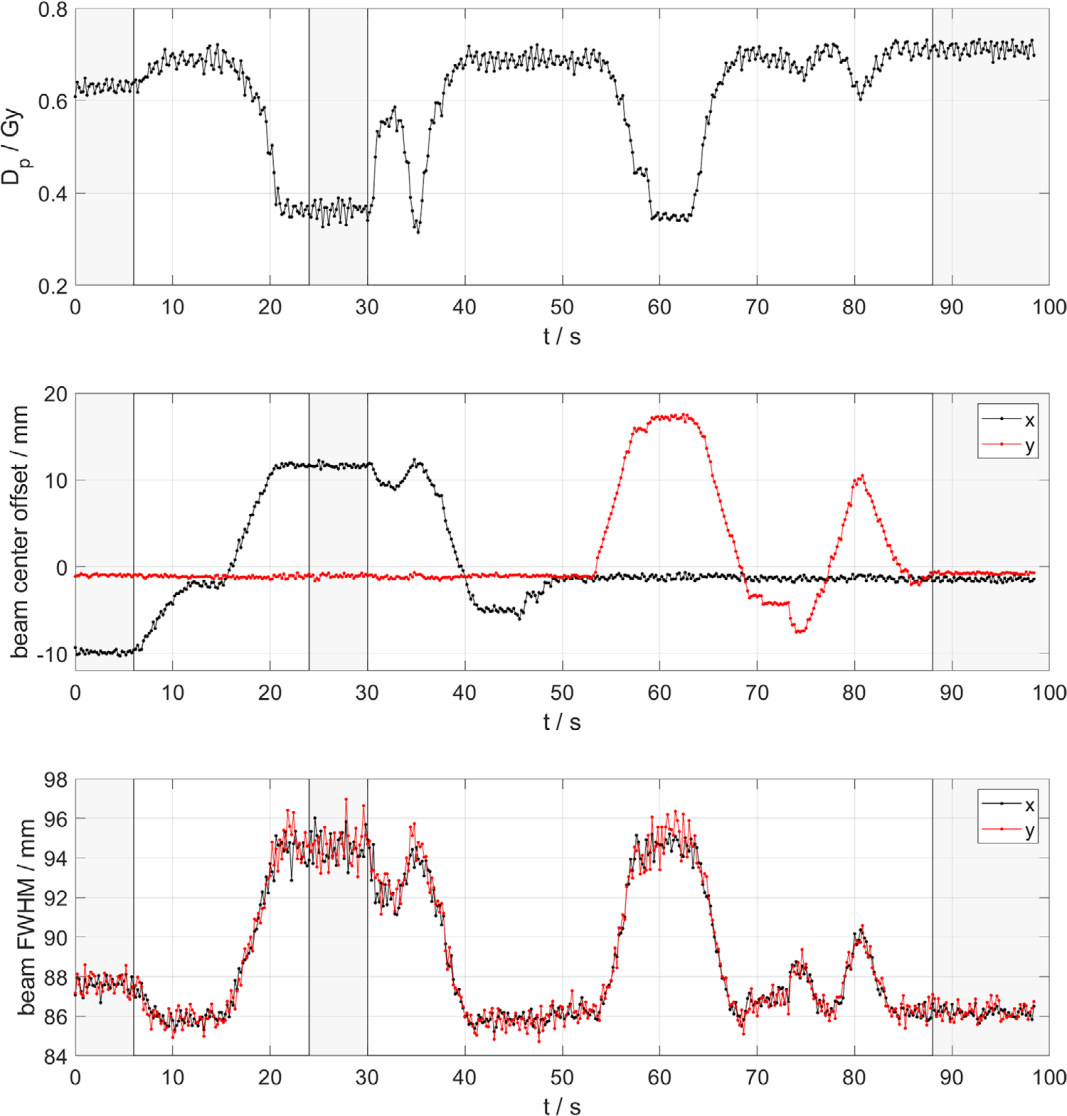
Time‐resolved measurement of beam characteristics using the FLASH Profiler during a beam steering process. Top panel: dose per pulse $D_{p}$ to the central diode of the FLASH profiler over time. Middle panel: beam center offset over time showing offset in *x*‐ and *y*‐directions in black and red respectively. Bottom panel: beam full‐width‐half‐maximum (FWHM) over time. Beam profiles corresponding to the shaded regions are shown in Figure 10. Each data point represents a single delivered pulse.

**FIGURE 10:**
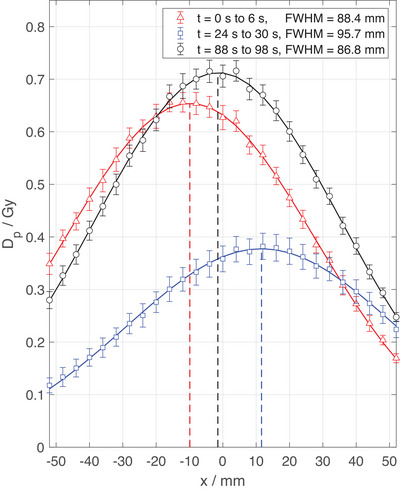
Averaged crossline beam profiles corresponding to the shaded regions in Figure 9. Dashed lines indicated the beam center positions on the *x*‐axis.

Given the ability of the FLASH Profiler to measure lateral beam profiles on a pulse‐by‐pulse basis at the accelerator's pulse repetition frequency, a beam steering process was simulated and the dose to the central diode, the beam center offset in *x*‐ and *y*‐directions and the beam's FWHM were measured in real time (Figure 9) over a total time of 98.4 s (492 pulses). Each data point represents a single delivered pulse. Exemplary crossline beam profiles are shown in Figure 10, which correspond to the shaded time intervals in Figure 9.

## 4 DISCUSSION

### 4.1 EDGE detector: Nonlinear response at high doses per pulse

Figures 3, 4, 5, 6 evaluate the performance of a single SunPoint 1 diode within the EDGE detector, which allowed for flexible variation and evaluation of electrical components connected to the diode. It is evident that the diode's response linearity is limited on both ends of the evaluated range of $D_{P}$, as can be seen in the bottom panel of Figure 3. Note that in Figure 3 the detector response curves at two different pulse lengths $t_{p}$ for $R_{C}=25\;\Omega$ (solid and open black markers) coincide, when the figure is plotted over instantaneous dose rate rather than dose per pulse. This is due to the voltage $U_{D}$ on the diode being a function of the ionization current and the series resistance $R_{S}+R_{C}$.

A sharp drop in response toward higher values of $D_{P}$ is observed for high series resistance $R_{S}+R_{C}$ between the diode and the capacitors $C_{C}+C_{B}$ (see values for $R_{C}=25\;\Omega$ in Figure 3) and also for small capacitances $C_{C}+C_{B}$ (see left panel of Figure 4). The series resistance $R_{S}+R_{C}$, as well as the capacitor size of $C_{C}+C_{B}$, affect the voltage $U_{D}$ on the diode during a pulse. According to the current‐voltage characteristics of the SunPoint 1 diode (right panel of Figure 4), the diode becomes conductive at a forward bias voltage of about 0.4 V.

This voltage coincides with the voltage $U_{D}$ on the diode at which the drop in response is observed in Figures 3 and 4. The interpretation is supported by the fast discharge process through the diode visible in panel b) of Figure 5, where a fast decay of charge is visible for capacitor voltage above 0.4 V. Notably, the discharge through the diode, which is conductive at forward bias voltages exceeding about 0.4 V (right panel of Figure 4), stops at about 0.4 V and the remaining charge is collected by the electrometer. This behavior also explains the capped diode response observed for a cable resistance of 25 Ω in Figure 3. Panel a) of Figure 5 shows the same data for a capacitor five times larger, which reduces $U_{D}$ by the same factor for a given ionization charge. Thus $U_{D}$ < 0.4 V up to 6 Gy per pulse (Figure 4).

In summary, the combination of a large series resistance and a small capacitor forward biases the diode above its forward junction voltage causing it to become conductive and lose a portion of charge generated in the diode. Other studies have shown similar diode behavior with a PTW microSilicon diode^34^ and a PTW microDiamond detector^30^ in UHPDR electron beams.

### 4.2 EDGE detector: Nonlinear response at lower doses per pulse

A response nonlinearity is also observed at $D_{P}$ < 1.5 Gy, where the response increases with increasing $D_{P}$. This effect has also been reported with a smaller magnitude for silicon carbide diodes in UHPDR electron beams^34^ and was further investigated by repeating the measurements with different electrometers in different measurement modes, with different EDGE detectors with varying levels of pre‐irradiation (0 Gy to > 1 MGy), with different cables, and by shielding the RC component against stray radiation. All tested configurations confirmed the observed response increase with increasing $D_{P}$.

Notably, the response of the EDGE detector without pre‐irradiation matched the nominal value of 32 nC·Gy^−1^ at $D_{P}$ < 0.2 Gy, while the response increased to 38·Gy^−1^ at $D_{P}$ = 1.5 Gy and remained constant for higher values of $D_{P}$. This suggests that the diode is entering the high‐injection mode described by Shi et al. at $D_{P}$ ≈ 1.5 Gy, which corresponds to an instantaneous dose rate of 750 kGy·s^−1^. High injection is reached when the recombination‐generation centers of a diode are almost full, therefore allowing constant recombination and, consequentially, a constant response with further increasing instantaneous dose rate.^53^

### 4.3 EDGE detector: Temporal response of the SunPoint 1 diode

The temporal response of the diode was tested and exemplary plots with a resolution of 4 ns are shown in Figure 6. Evidently, the SunPoint 1 diode is capable of measuring the pulse length as well as the intra‐pulse dose rate fluctuations. Both the EDGE Detector and the ICT measured pulse lengths of $t_{p}=$ 1.85 µs (left panel of Figure 6), and $t_{p}=$ 0.97 µs (right panel of Figure 6). The high‐frequency oscillations observed after the pulse are likely caused by dielectric components and the low‐frequency oscillations are due to a non‐optimized electrical connection (impedance mismatch) between the diode and the oscilloscope in this setup. The measurable range of $D_{P}$ in this setup was limited by the resistor $R_{M}$, over which the voltage drop was measured, where a smaller resistor would allow for higher doses per pulse at the cost of more prominent charge oscillation. The bottom panel of Figure 6 confirms that their net effect on accumulated charge is insignificant. Similar results were presented for a silicon carbide diode in UHPDR beams^34^ and fD,^54^ where the SunPoint 1 diode has the advantage of being manufacturable in a detector array arrangement.

While the pulse repetition frequency in this study is low compared to some (pre‐)clinical UHPDR TDDs^15^, ^16^, ^17^ or modified clinical linear accelerators,^11^, ^12^, ^13^, ^14^ the speed of response (top panels of Figure 6) and charge collection time (bottom panels of Figure 6) observed for the SunPoint 1 Diode allow for significantly higher pulse repetition frequencies. Rahman et al. experimentally demonstrated the applicability of the SunPoint 1 Diode in UHPDR electron beams with dose rates of up to 180 Gy/s using pulse repetition frequencies of 60–360 Hz.^29^

### 4.4 FLASH profiler

The consideration of series resistance and the capacitance of the RC unit is specifically important for the SunPoint 1 diode since its nominal response of 32 nC·Gy‐1 is relatively high, that is about 100 times higher than that of the fD. According to the findings from Figures 3, 4, 5, an RC unit was implemented into the FLASH Profiler, which minimizes the series resistance between the SunPoint 1 diodes and the capacitor $C_{B}$ and provides a sufficiently large capacitance $C_{B}$ to store charge generated by a diode exposed to $D_{p}\;\leq$ 8 Gy. A resistor $R_{B}$ was implemented to prevent oscillation of charge between the capacitive components of the circuit.

The additional application of reverse bias voltage^51^ of 0.6 V significantly improves the response nonlinearity at $D_{P}$ < 1.5 Gy, as shown in the bottom panel of Figure 7. In addition, the $D_{P}$ range increases, since effectively a dose pulse generated junction voltage of 1 V may be generated before the forward junction voltage of 0.4 V is reached. Note that in Figure 7, the signal noise at low values of $D_{P}$ is due to the limited bit resolution of the AD converter. Consequentially, Figure 7 presents the FLASH Profiler's linearity of response to be within 3% for $D_{P}$ < 1 Gy and within ± 2% for $D_{p}\;>\;1$ Gy. No dependence of the response on the pulse length at equal instantaneous dose rate is apparent. While Figure 7 presents a detailed analysis of only the FLASH Profiler's central diode, the comparison to scanned beam profiles in Figure 8 indicates equivalent results for all other diodes of the array.

The SunPoint 1 diode's response degrades by up to 1.5% per kGy in electron beams,^55^ where the actual rate depends on the accumulated dose to the detector and the radiation quality.^56^ In this study, a degradation rate of about 0.3% per kGy was measured and corrected for. The effect necessitates recurrent homogenization of the array detectors’ response, which is achieved by the Wide Field Array Calibration^52^ as demonstrated in this study. Notably, all data points of a beam profile in Figure 8 were measured simultaneously, while the reference profiles were measured step‐by‐step with a fD. Thereby, the required delivered dose to measure full beam profiles is reduced to that of a single pulse. As a result, pulse‐by‐pulse analysis of beam profile characteristics, as well as beam steering are enabled, as shown in Figures 9 and 10.

While the numeric results presented in this study are specific to the SunPoint 1 diode, the observed behavior of the diode with respect to voltage $U_{D}$, capacitance $C_{C}+C_{B}$ and series resistance $R_{S}+R_{C}$ are transferrable to other diodes following a voltage‐current‐characteristics as shown in the right panel of Figure 4. The magnitude of the effects may depend on the diode construction, diode material, doping, or electrification, which can vary significantly among different diode designs. Consequently, the individual combination of diode type, cabling, RC unit, and electrometer will have to be optimized for use in UHPDR beams.

## 5 CONCLUSION

The FLASH Profiler can be used for characterizing lateral beam profiles of UHPDR electron beams in real‐time and on a single pulse basis. This can facilitate quality control and beam steering service of electron UHPDR TDDs at significantly reduced dose exposure, setup time, measurement time and cost.

## CONFLICT OF INTEREST STATEMENT

A.A.S., J.H., V.F., J.K., and W.S. are employees of Sun Nuclear Corp. The remaining authors declare that the research was conducted in the absence of any commercial or financial relationships that could be construed as a potential conflict of interest.

## Data Availability

The data that support the findings of this study are available from the corresponding author upon reasonable request.
